# Anaemia at antenatal care initiation and associated factors among pregnant women in West Gonja District, Ghana: a cross-sectional study

**DOI:** 10.11604/pamj.2019.33.325.17924

**Published:** 2019-08-27

**Authors:** Basil Addayire Tibambuya, John Kuumuori Ganle, Muslim Ibrahim

**Affiliations:** 1 Department of Public Health, West Gonja Hospital, Damongo, Northern Region, Ghana; 2 Department of Population, Family and Reproductive Health, School of Public Health, University of Ghana, Accra, Ghana; 3 Stellenbosch Institute for Advanced Study (STIAS), Wallenberg Research Centre at Stellenbosch University, Stellenbosch 7600, South Africa; 4 Nadowli Hospital, Ghana Health Service, Nadowli, Upper West Region, Ghana

**Keywords:** Anaemia, pregnancy, preconception care, early antenatal care, antenatal care initiation, iron-rich food, Ghana

## Abstract

**Introduction:**

Anaemia in pregnancy remains a critical public health concern in many African settings; but its determinants are not clear. The purpose of this study was to assess anaemia at antenatal care initiation and associated factors among pregnant women in a local district of Ghana.

**Methods:**

A facility-based cross-sectional survey was conducted. A total of 378 pregnant women attending antenatal care at two health facilities were surveyed. Data on haemoglobin level, helminths and malaria infection status at first antenatal care registration were extracted from antenatal records booklets of each pregnant women. Questionnaires were then used to collect data on socio-demographic and dietary variables. Binary and multivariate logistic regression analyses were done to assess factors associated with anaemia.

**Results:**

The prevalence of anaemia was 56%, with mild anaemia being the highest form (31.0%). Anaemia prevalence was highest (73.2%) among respondents aged 15-19 years. Factors that significantly independently reduced the odds of anaemia in pregnancy after controlling for potential confounders were early (within first trimester) antenatal care initiation (AOR=5.01; 95% CI =1.41-17.76; p=0.013) and consumption of egg three or more times in a week (AOR=0.30; 95% CI=0.15-0.81; P=0.014).

**Conclusion:**

Health facility and community-based preconception and conception care interventions must not only aim to educate women and community members about the importance of early ANC initiation, balanced diet, protein and iron-rich foods sources that may reduce anaemia, but must also engage community leaders and men to address food taboos and cultural prohibitions that negatively affect pregnant woman.

## Introduction

Globally, anaemia affects an estimated 43% of children, 38% of pregnant women, and 29% of non-pregnant women of childbearing age [1]. In low-income countries, anaemia affects 40 to 60% of pregnant women [2, 3]. The World Health Organization defines anaemia as decreased concentration of haemoglobin (Hb) level of less than 11g/dL [1]. Anaemia during pregnancy is considered severe when Hb concentration level is less than 7.0g/dL; moderate when haemoglobin level falls between 7.0-9.9g/dL; and mild from 10.0-10.9g/dL [4]. The causes of anaemia during pregnancy are multi-factorial, and includes nutritional deficiencies of iron, folate, and vitamin B12 [1]. Economic and socio-cultural factors such as cultural and religious food taboos also significantly contribute to anaemia among pregnant women [3, 5]. Other causes of anaemia in pregnancy include parasitic infections like helminths and other conditions such as low intake or poor absorption of iron [4]. Iron deficiency is the most common cause of anaemia in pregnancy in many low-income settings [2, 4]. While evidence suggests that most women in low-income countries, including Ghana, enter pregnancy with less than adequate stores of nutrients [6], anaemia in pregnant women could have serious adverse pregnancy outcomes, including high maternal death, impaired mental development in children, increased risk of fetal growth retardation, low birth weight, premature delivery and perinatal mortality [7, 8]. Like many countries in Africa, anaemia remains an important threat to safe motherhood and newborn health in Ghana [9, 10]. Anaemia is the number two cause of all admissions and the number five cause of death among all admitted patients in Ghana [11]. Indeed, health facility level data suggest that the prevalence of anaemia among pregnant women in Ghana is on the rise, from 34% in 2014 to 37% in 2016 [11]. There are however regional disparities. In the Northern region where this study was conducted, 43.2% of pregnant women attending ANC in 2016 were anaemic [11]. The situation in the specific district (West Gonja District) where this study was conducted is worse: anaemia among ANC attendants rose from 23.4% in 2012 to 43.9% in 2016 [12]. While the potential adverse health consequences of anaemia in pregnancy are widely recognised, few empirical studies have been conducted in Ghana to identify key determinants [10]. Indeed, the lack of evidence on anaemia in many low-income countries is acknowledged as one of the reasons why the fight against anaemia in pregnancy still remains a problem [1]. This study aimed to assess anaemia at antenatal care initiation and its determinants among pregnant women in a local district of Ghana.

## Methods

**Study design and respondents:** a facility based cross-sectional quantitative survey was conducted at the west Gonja District Hospital and the Damango Health Centre, all in the West Gonja District of the Northern region of Ghana between November 2017 and April 2018. All pregnant women aged 15-49 years who were attending these two health facilities to receive their first ANC between November 2017 and April 2018 were eligible for the study. However, pregnant women who reported a recent history of blood transfusion (within the past three months) before initiation of first ANC were excluded from the study.

**Study setting:** the west Gonja District has an estimated population of 49,386 [13]. Women form 51% of the district's population, with about 1,975 women expected to have become pregnant in 2017 [12]. The main occupations of women in the district are farming and retail trade and services, with few engaged in teaching and nursing [12]. Health service delivery in the district is done through a total of thirteen (13) community-based health planning and services (CHPS) compounds, five (5) health centres, and one (1) district hospital. All the 19 facilities provide basic ANC services. However, the West Gonja Hospital (the main referral hospital) provides comprehensive prenatal, delivery and postnatal services. The West Gonja hospital and Damongo Health Centre (the largest first-tier primary public healthcare facility) were purposively selected for this study. These facilities are the largest public health facilities and receive the largest number of ANC registrants on annual basis.

**Sample size:** a total of 433 pregnant women reported for their first ANC in the two facilities (224 in West Gonja hospital and 209 in Damongo health centre) between November 2017 and April 2018. However, 34 women had history of recent blood transfusion and 21 women who met the inclusion criteria declined to participate. They were therefore excluded from the study, leaving a final sample size of 378.

**Recruitment and data collection:** all respondents were recruitment at the ANC clinics of the two health facilities. Two research assistants were trained and stationed at each of the two ANC clinics. Starting from November 1, 2017 to April 30, 2018, the research assistants attended all weekly ANC clinic sessions organised by midwives/nurses. Pregnant women who reported to the clinics for their first ANC were all approached after they (women) had completed all service procedures and were exiting. They were individually told about the purpose of the study and the study procedures. Those who could read (in English) were immediately provided with information leaflets about the study. Those who could not read were asked if they wanted to receive the information leaflet so that a family member or friend could later read and explain to them. Nearly all such women accepted the information leaflets. The research assistants enlisted the names and contact numbers of all the women approached. Those without personal telephone numbers were requested to provide the numbers of their husband/partner, family member or friend. Following from this, each woman was given two weeks to decide on their participation. They were each re-contacted via telephone after the two-week period. Where the decision was in favor of participation, interview dates were arranged, usually on the next ANC visit. However, where the decision was against participation (there were 21 such cases), such women were dropped.

In terms of data collection, two methods were employed: data extraction from ANC booklets of respondents and administration of structured questionnaires. First, the following information was extracted from the ANC booklet: timing of ANC initiation, Hb level at registration, helminths infection, malaria infection, number of times the woman became pregnant and number of children delivered by the woman. HB level, helminths infection and malaria infection are routine blood tests done for all pregnant women at ANC initiation. A simple tool was designed and used to extract this information from the maternal and child health record books of each of the 378 women who agreed to participate in the study. This information was then subsequently linked to information collected from each woman using the questionnaires. Second, questionnaires were used to collect data on other socio-demographic, maternal and dietary characteristics. The questionnaires were pre-tested at two other smaller health centres located in the district. All necessary corrections were made before actual data collection from November 2017 to April 2018. Actual data collection occurred alongside recruitment: as women reported to the ANC clinic on weekly basis for the first time, they were approached, recruited and subsequently interviewed. The two research assistants conducted all interviews in a designated small room within the premises of each health facility. Women were interviewed one at a time. English and Gonja (local dialect) were the interview languages.

**Data entry and processing:** completed questionnaires were manually examined for completeness, then hand-coded and entered into Epi info version 7. The data were independently entered by the two research assistants. The first and second authors then independently compared the two data entries. All errors were discussed and resolved before data were exported into Stata (version 15.0) for further cleaning and analysis.

**Variables:** the main outcome variable is anaemia, which we defined and measured primarily as a binary outcome. We followed the WHO's definition and categorisation: women whose haemoglobin (Hb) concentration levels were >11g/dl and <11g/dl were classified as 'not anaemic' and 'anaemic' respectively [1]. We re-categorized all anaemic women into mild (10-10.9g/dl), moderate (7-9.9g/dl) and severe (<7g/dl). Several independent variables were also defined and measured, including socio-demographic factors such as age, maternal education, occupation, marital status, religion, husband's occupation, and place of residence as well as maternal and dietary characteristics. Timing of ANC initiation was determined by whether the woman came within the first, second or third trimester. Malaria infection was determined by whether a woman tested Positive or Negative for the presence of malaria parasite at the time of ANC initiation. Helminthic infection was also determined by whether the woman tested Positive or Negative for any intestinal worm infection during her current pregnancy at the time of ANC initiation.

**Statistical analysis:** categorical variables were summarised into frequencies and proportions. Continuous variables were summarised into means and ranges and continuous variables like age were re-categorised into age groups. Bivariate analysis was first done using chi-square test of independence to assess association between anaemia and categorical independent variables. Binary logistic regression was used to assess for factors associated with anaemia. Factors with p-value < 0.05 at 95% confidence level were considered statistically significant and were therefore included in a multiple logistic regression model for further analysis. Odd ratios were estimated.

**Ethical considerations:** the research was conducted in accord with prevailing ethical principles. Ethical approval was obtained from the Ghana Health Service Ethical Review Committee (GHS-ERC Number: GHS-ERC 20/02/2017). Informed written consent was obtained (either by signing or thumb printing) from each respondent before interviewing.

## Results

**Characteristics of respondents:** Table 1 shows the background characteristics of the 378 respondents who took part in the study. The mean age was 26.9, and the majority (29.1%) were aged 25-29 years. Table 2 also shows the maternal characteristics of respondents. Majority (51.9%) initiated ANC in the second trimester (13 to 24 weeks). Some 13.8% of the respondents tested positive for malaria at their first ANC visit, while 31.2% tested positive for helminths infection. Table 3 describes the dietary characteristics of respondents. A combined 55.8% of the respondents took meat (including liver) and fish at least three times a week. Some 51.1% also consumed egg 1-2 times per week. Green leafy vegetable consumption was generally high among respondents: 21.4% and 76.7% consumed green leafy vegetable 1-2 times and 3+ times per week respectively.

**Table 1:** Demographic and economic characteristics

Characteristic	Frequency (n=387)	Percent
**Mother’s Age**		
**Mean age (SD)**	26.9+10.1	
15-19	41	10.9
20-24	104	27.5
25-29	110	29.1
30-34	75	19.8
35+	48	12.7
**Mother’s Education**		
None	131	34.7
Primary	57	15.1
Junior High School (JHS)	85	22.5
Secondary	65	17.2
Tertiary	40	10.6
**Marital Status**		
Cohabitation	11	2.9
Divorced	1	0.3
Married	315	83.3
Single	51	13.5
**Religious Affiliation**		
Christianity	91	24.1
Islam	285	75.4
Traditional	2	0.5
**Place of Residence**		
Rural	163	43.1
Urban	215	56.9
**Mother’s Occupation**		
Government worker	28	7.4
Self-employed	195	51.6
Unemployed	155	41.0
**Distance to Facility for ANC (km)**		
<1	139	36.8
2-4	142	37.6
5-7	79	20.9
8-10	18	4.8
**Monthly Expenditure (GH¢)**		
<200	278	73.5
200-500	89	23.5
600-1,000	9	2.4
1,100+	2	0.5
**Monthly earnings (GH¢)**		
<200	257	68.0
200-500	98	25.9
600-1,000	16	4.2
1,100+	7	1.9
**Partner’s Education Level**		
None	132	34.9
Primary	37	9.8
JHS	52	13.8
Secondary	80	21.2
Tertiary	77	20.4
**Partner’s Occupation**		
Government worker	73	19.3
Self-employed	233	61.6
Unemployed	72	19.1

**Table 2:** Maternal characteristics

Characteristic	Frequency (n=387)	Percent
Timing of ANC Initiation		
**First trimester**	161	42.6
**Second trimester**	196	51.9
**Third trimester**	21	5.6
Parity		
**0-4**	341	90.7
**5+**	35	9.3
Gravidity		
**1- 4**	337	89.2
**5+**	41	10.9
Birth Spacing (years)		
**1**	15	4.0
**2+**	272	72.0
**Primigravida**	91	24.0
Ownership of Treated Bed Net		
**Yes**	360	95.2
**No**	18	4.8
Sleep Under Treated Bed Net Everyday		
**Yes**	331	87.6
**No**	47	12.4
Malaria Infection at ANC Initiation		
**Yes**	81	21.5
**No**	297	78.5
Helminths Infection at ANC Initiation		
**Yes**	118	31.2
**No**	260	68.8

**Table 3:** Dietary and cultural characteristics

Characteristic	Frequency (n=387)	Percent
**Lipton/Coffee Tea Consumption (at least once a week)**		
Yes	176	46.6
No	202	53.4
Meat/Fish Consumption Per Week*		
None	15	4.0
1-2 times	152	40.2
3+ times	211	55.8
**Egg Consumption Per Week**		
None	56	14.8
1-2 times	193	51.1
3+ times	129	34.1
**Green leafy Vegetable Consumption Per Week**		
None	7	1.9
1-2 times	81	21.4
3+ times	290	76.7
**Food Prohibited during Pregnancy**		
Egg	15	4.0
Meat	11	2.9
None	352	93.1

* The purpose for measuring meat(liver) and fish together was to assess meat-based sources of iron

**Prevalence of anaemia:** in terms of prevalence of anaemia, 55.8% of the respondents were anaemic (Hb less than 11g/dl), with the mean Hb level being 10.8g/dl and a range of 6.7g/dl to 14.4g/dl. Among the 55.8% who had anaemia, 0.3% had severe anaemia, 24.5% had moderate anaemia, and 31.0% had mild anaemia.

**Predictors of anaemia:** to determine factors associated with anaemia in pregnancy, chi-square tests of independence were first performed between a total of 24 independent variables and anaemia in pregnancy. From this initial analysis, 11 factors were statistically associated with anaemia in pregnancy. These 11 factors were then pulled into binary and multiple logistic regression models and odds ratios were estimated. The results are shown in Table 4 and Table 4 (suite). After adjusting for potential confounders, two factors significantly independently predicted anaemia in pregnancy: timing of ANC initiation and egg consumption per week. Women who initiated ANC within the second and third trimesters were, respectively, 2.71 and 5.01 times more likely to be anaemic compared to those who started ANC within the first trimester (AOR=2.71; 95% CI=2.09-5.81; P<0.01) and (AOR=5.01; 95% CI =1.41-17.76; p=0.013). The odds of getting anaemia in pregnancy significantly declined as a pregnant woman consumed eggs more frequently per week. When compared to women who reported not consuming egg at all, the odds of being anaemic in pregnancy were 0.51 lower for women who consumed egg 1-2 times per (AOR=0.51; 95% CI=0.29-1.39; p=0.257), and 0.30 times lower for women who consumed egg 3+ times per week (AOR=0.30; 95% CI=0.15-0.81; P=0.014).

**Table 4:** Predictors of anaemia in pregnancy (multivariable logistic regression analysis)

Characteristic	Anaemic, n (%)	Not Anaemic, n (%)	Unadjusted OR (95%CI)	P-value	Adjusted OR (95%CI)	P-value
**Mother’s age**						
15-19 (ref)	30(73.2)	11(26.8)	1		1	
20-24	64(61.5)	40(38.5)	0.59(0.26-1.30)	0.189	0.99(0.37-2.70)	0.992
25-29	60(54.6)	50(45.5)	0.44(0.20-0.97)	0.041*	1.02(0.35-2.97)	0.971
30-34	37(49.3)	38(50.7)	0.36(0.16-0.82)	0.015*	0.69(0.22-2.17)	0.522
35+	20(41.7)	28(58.3)	0.26(0.11-0.64)	0.003*	0.37(0.11-1.22)	0.102
**Mother’s Education**						
Tertiary (ref)	13(32.5)	27(67.5)	1		1	
Secondary	34(52.3)	31(47.7)	2.28(1.00-5.18)	0.049*	0.44(0.12-1.61)	0.216
JHS	48(56.5)	37(43.5)	2.69(1.23-5.93)	0.014*	0.40(0.10-1.57)	0.187
Primary	36(63.2)	21(36.8)	3.56(1.52-8.35)	0.004*	0.68(0.16-2.78)	0.586
None	80(61.1)	51(38.9)	3.26(1.54-6.89)	0.002*	0.80(0.20-3.29)	0.762
**Marital Status**						
Single (ref)	38(74.5)	13(25.5)	1		1	
Married	165(52.4)	150(47.6)	0.38(0.19-0.73)	0.004*	0.50(0.21-1.20)	0.122
Cohabitation	7963.6)	4(36.4)	0.60(0.15-2.38)	0.466	0.52(0.11-2.59)	0.427
Divorced	1(100.0)	0(0.0)**				
**Mother’s Occupation**						
Unemployed (ref)	101(65.2)	54(34.8)	1		1	
Self-employed	103(52.8)	92(47.2)	0.60(0.39-0.92)	0.020*	0.89(0.50-1.59)	0.700
Government worker	7(25.0)	21(75.0)	0.18(0.07-0.45)	0.000*	0.36(0.08-1.77)	0.210
**Monthly Expenditure (GH¢)**						
<200 (ref)	171(61.5)	107(38.5)	1		1	
200-500	38(42.7)	51(57.3)	0.47(0.29-0.76)	0.002*	1.27(0.53-3.03)	0.588
600-1,000	2(22.2)	7(77.8)	0.18(0.04-0.88)	0.034*	1.45(0.16-3.52)	0.744
1,100+	0(0.0)	2(100.0)**				
**Monthly Earnings (GH¢)**						
<200	162(63.0)	95(37.0)	1		1	
200-500	3(18.8)	13(81.3)	0.48(0.30-0.77)	0.002*	0.65(0.29-1.44)	0.288
600-1,000	44(44.9)	54(55.1)	0.14(0.04-0.49)	0.002*	0.21(0.04-1.16)	0.074
1,100+	2(28.6)	5(71.4)	0.23(0.05-1.23)	0.087	1.69(0.12-2.93)	0.693

* p<0.05; OR= odds ratio; CI=confidence interval; *ref*=reference categories

** Marital Status! =0 predicts success perfectly, hence marital status was dropped and 1 observation not used. Also, monthly expenditure! =0 predicts failure perfectly, hence monthly expenditure was dropped and 2 observations not used

**Table 4 (suite):** Predictors of anaemia in pregnancy (multivariable logistic regression analysis)

Characteristic	Anaemic, n (%)	Not Anaemic, n (%)	Unadjusted OR (95%CI)	P-value	Adjusted OR (95%CI)	P-value
**Partner’s Education**						
Tertiary *(ref)*	29(37.7)	48(62.3)	1		1	
Secondary	43(53.8)	37(46.2)	1.92(1.02-3.64)	0.044*	1.85(0.55-6.51)	0.339
JHS	39(75.0)	13(25.0)	4.97(2.28-10.82)	0.000*	3.89(0.89-17.07)	0.072
Primary	26(70.3)	11(29.7)	3.91(1.69-9.08)	0.002*	3.59(0.76-16.89)	0.106
None	74(56.1)	58(43.9)	2.11(1.19-3.75)	0.011*	1.48(0.36-6.11)	0.590
**Partner’s Occupation**						
Unemployed *(ref)*	44(61.1)	28(38.9)	1		1	
Self-employed	137(58.8)	96(41.2)	0.91(0.53-1.56)	0.727	0.69(0.35-1.38)	0.293
Government worker	30(41.1)	43(58.9)	0.44(0.23-0.86)	0.017*	1.48(0.40-5.47)	0.557
Timing of ANC Initiation						
First trimester *(ref)*	66(41.0)	95(59.0)	1		1	
Second trimester	128(65.3)	68(34.7)	2.71(1.76-4.17)	0.000*	3.49(2.09-5.81)	0.000*
Third trimester	17(81.0)	4(19.0)	6.12(1.97-19.01)	0.002*	5.01(1.41-17.76)	0.013*
**Birth Spacing (yrs)**						
1 *(ref)*	11(73.3)	4(26.7)	1		1	
2+	139(51.1)	133(48.9)	0.38(0.12-1.22)	0.105	0.53(0.14-2.08)	0.368
Primigravida	61(67.0)	30(33.0)	0.74(0.22-2.52)	0.629	0.84(0.20-3.42)	0.804
**Egg Consumption per Week**						
Never *(ref)*	41(73.2)	15(26.8)	1		1	
1-2 times	112(58.0)	81(42.0)	0.51(0.26-0.96)	0.042*	0.63(0.29-1.39)	0.257
3+ times	58(45.0)	71(55.0)	0.30(0.15-0.59)	0.001*	0.35(0.15-0.81)	0.014

* p<0.05; OR= odds ratio; CI=confidence interval; *ref*=reference categories

## Discussion

This study is one of the few to assess anaemia prevalence and associated factors among pregnant women attending ANC services in Ghana. Results suggest that the prevalence of anaemia among pregnant women in the study is quite high (56%), with mild anaemia being the highest (31.0%) form. Two factors significantly independently predicted anaemia in pregnancy after adjusting for other factors, namely timing of ANC initiation and egg consumption per week. Several aspects of these results deserve further reflection on. The prevalence of anaemia in this study is highest (73.2%) among respondents aged between 15-19 years. This is consistent with findings from Mangla & Singla's study [6]. A number of factors could contribute to high anaemia in this age group. One of the important causes of anaemia is iron deficiency, and studies suggest that the 15-19year age band is a period of intense physical and mental growth, with a higher demand for iron and other nutrients [4, 10]. Pregnancy and childbirth during this age group could place further demands on the already inadequate iron stores in teenage mothers. This could easily predispose pregnant teenagers to anaemia. Apart from the fact that young girls may be unprepared biologically, they may also be unprepared emotionally and economically to deal with pregnancy. This is particularly likely because in many contexts in Ghana, sexual and reproductive health topics remain taboo subjects for most parents to discuss with their adolescent children, and teen pregnancy is often not welcome [14]. This could easily undermine social and economic support for teenage mothers, which could in turn affect their nutritional status. This would suggest a need to intensify early sexual and contraception education and counselling for female adolescents at home and in school as well as self-efficacy training and skills acquisition to help them negotiate peer-pressures to initiate sex early and to protect themselves during sexual intercourse. The role of parents and guardians in providing sexual and reproductive health education needs to be encouraged given that early sexual debut and childbearing among female adolescents is a widely reported phenomena in Africa [15-17]. Apart from interventions to stop or reduce early sexual debut and childbearing, interventions to encourage teen mothers to seek early ANC together with targeted nutritional counselling and support services, would also be essential.

The timing of ANC initiation emerged as an important predictor of anaemia among first time registrants. Compared to women who initiated ANC in the first trimester, the odds of having anaemia in pregnancy were still significantly higher among pregnant women who initiated ANC in the second trimester (AOR=2.71; 95% CI=2.09-5.81; P<0.01) and third trimester (AOR=5.01; 95% CI=1.41-17.76; p=0.013). This is also consistent with what has been reported in Bangladesh [18], and in a regional health facility study in South Africa [8], where anaemia in pregnancy was higher among women who registered in the second and third trimesters compared to those who registered within the first trimester. That late ANC initiation is associated with anaemia in pregnancy is however not surprising. This is not only because majority of women in our study initiated ANC either in the second or third trimester, but also because late ANC initiation means that many of the interventions and services routinely offered to pregnant women at ANC clinics to prevent anaemia in pregnancy such as IFA supplementation, provision of LLINs, and IPT dosing, as well as laboratory investigations (e.g. Hb check and stool tests) to diagnose early anaemia in pregnancy and offer early treatment, are delayed for such women. This would suggest a need for both health facility and community-based preconception and conception care interventions to educate women and community members on the importance of early antenatal care initiation and the need to seek ANC services early. In doing this, efforts must be made to address health system barriers such as long distances to service centres as well as engage community members (men and mothers-in-law in particular) to address socio-cultural barriers such as the need to perform traditional pregnancy-related rituals before permission is granted for pregnant women to access services as shown in previous research in northern Ghana [19, 20].

Some aspects of dietary characteristics were also significantly associated with anaemia in pregnancy. While meat/fish and green leafy vegetable consumption did not surprisingly show significant statistical association with anaemia in pregnancy as we expected, frequency of egg consumption did show strong statistical association with anaemia such that women who consumed eggs three or more times in a week were less likely to be anaemic in pregnancy compared to those who did not consume eggs at all. This is similar to findings by Gebre & Mulugeta in northern Ethiopian where frequency of egg consumption per day was strongly associated with anaemia in pregnancy [3]. Our results here support existing evidence that highlights the nutritional benefits of egg consumption (at least in the context of anaemia) during pregnancy and suggest a need for pregnant women to incorporate eggs into their diet. We acknowledge that poverty and socio-cultural food laws and taboos could deny pregnant women otherwise nutritionally rich food sources including meat and egg. Nutrition supplementation and community-based interventions to address harmful food taboos during pregnancy could be essential. Taken together, this study has provided important insights into the anaemia and dietary situation among pregnant women who started ANC between November 2017 and April 2018 in the west Gonja District. The findings give an indication of the factors that may be contributing to anaemia in pregnancy. This could potentially afford policy makers and healthcare workers an opportunity to plan and implement contextually relevant interventions to reduce anaemia and its associated adverse consequences. The findings also provide a basis for large-scale further quantitative and qualitative studies in different contexts in Ghana to estimate anaemia prevalence, identify important determinants and explore detailed contextual, structural and personal level explanatory factors. The study however has some limitations. First, the study only assessed anaemia at registration and did not examine anaemia at various stages of pregnancy (e.g. anaemia at 28 weeks and at 36 weeks). Such an analysis could provide better understanding on the prevalence at each stage. Second, data on Hb level, malaria and helminths infection were extracted from maternal and child health records of each woman. Any original data errors resulting from inaccurate test results or improper data capture could not have been addressed. Finally, there could be recall bias since respondents were asked about dietary and other behaviours that might have taken place long before this study.

## Conclusion

The main objective of this study was to assess anaemia prevalence and associated factors among pregnant women attending ANC services in the West Gonja District. The study revealed a relatively high (56%) anaemia prevalence among the study respondents. Timing of ANC and regular egg consumption were the strongest predictors of anaemia in pregnancy. These findings and discussion together suggest that awareness and knowledge about anaemia among pregnant women attending ANC alone may not even be sufficient to bring about reduced prevalence. Therefore, interventions need to go beyond awareness and knowledge creation through information provision to focusing on other important dietary, economic and cultural factors that may impact negatively on the possibility of getting anaemia in pregnancy. In this regard, health facility and community-based preconception and conception care interventions must not only aim to educate women and community members on the importance of early ANC initiation, balanced diet and sources of iron rich foods that may reduce anaemia, but must also engage community leaders to address issues related to food taboos and prohibitions during pregnancy that could expose pregnant women to adverse health outcomes, including anaemia.

### What is known about this topic

- Anaemia affects 40-60% of pregnant women in low-income countries;
- Anaemia is a significant contributory factor to adverse pregnancy outcomes, including high maternal death, impaired mental development in children, increased risk of fetal growth retardation, low birth weight, premature delivery and perinatal mortality;
- But its determinants are not exactly clear.

### What this study adds

- Early (within first trimester) antenatal care initiation and consumption of egg three or more times in a week significantly independently reduced the odds of being anaemic in pregnancy;
- Preconception and conception care interventions must stress the importance of early antenatal care initiation, consumption of balanced diet and protein and iron-rich foods;
- Community-based engagement and interventions to address food taboos and cultural prohibitions that negatively affect pregnant women are needed.

## Competing interests

The authors declare no competing interests.

## References

[1] World Health Organization (2015). The Global Prevalence of Anaemia in 2011.

[2] Balarajan Y, Ramakrishnan U, Özaltin E, Shankar AH, Subramanian SV (2011). Anaemia in low-income and middle-income countries. Lancet.

[3] Gebre A, Mulugeta A (2015). Prevalence of anemia and associated factors among pregnant women in north western zone of Tigray, Northern Ethiopia: a cross-sectional study. Journal of Nutrition and Metabolism.

[4] Obai G, Odongo P, Wanyama R (2016). Prevalence of anaemia and associated risk factors among pregnant women attending antenatal care in Gulu and Hoima Regional Hospitals in Uganda: a cross sectional study. BMC Pregnancy and Childbirth.

[5] Dattijo LM, Daru PH, Umar NI (2016). Anaemia in Pregnancy: prevalence and associated factors in Azare, North-East Nigeria. International Journal of Tropical Disease & Health.

[6] Mangla M, Singla D (2016). Prevalence of anaemia among pregnant women in rural India: a longitudinal observational study. International Journal of Reproduction, Contraception, Obstetrics and Gynecology.

[7] Campigotto AC, Duarte de Farias A, Ferreira Pinto DC, Albuquerque FG (2015). Factors relating to iron deficiency anemia in pregnancy: an integrative Review. International Archives of Medicine.

[8] Tunkyi K, Moodley J (2016). Prevalence of anaemia in pregnancy in a regional health facility in South Africa. South African Medical Journal.

[9] Browne ENL, Maude GH, Binka FN (2001). The impact of insecticide-treated bednets on malaria and anaemia in pregnancy in Kassena-Nankana district, Ghana: a randomized controlled trial. Tropical Medicine and International Health.

[10] Anlaakuu P, Anto F (2017). Anaemia in pregnancy and associated factors: a cross sectional study of antenatal attendants at the Sunyani Municipal Hospital, Ghana. BMC Research Notes.

[11] Ghana Health Service (2016). Annual Health Report 2016.

[12] West Gonja District Health Administration (2018). Half-Year Health Performance Review Report.

[13] Ghana Statistical Service (2014). Population and Housing Census 2010: District Analytical Report, West Gonja District.

[14] Nyarko SH (2015). Prevalence and correlates of contraceptive use among female adolescents in Ghana. BMC Women's Health.

[15] Rijsdijk LE, Bos AE, Lie R, Ruiter RA, Leerlooijer JN, Kok G (2012). Correlates of delayed sexual intercourse and Condom use among adolescents in Uganda: a cross-sectional study. BMC Public Health.

[16] Eliason S, Awoonor-Williams JK, Eliason C, Novignon J, Nonvignon J, Aikins M (2014). Determinants of modern family planning use among women of reproductive age in the Nkwanta district of Ghana: a case-control study. Reproductive Health.

[17] Babatunde OA, Ibirongbe DO, Omede O, Babatunde OO, Durowade KA, Salaudeen AG (2016). Knowledge and use of emergency contraception among students of public secondary schools in Ilorin, Nigeria. Pan African Medical Journal.

[18] Chowdhury AH, Ahmed RK, Jebunessa F, Akter J, Hossain S, Shahjahan M (2015). Factors associated with maternal anaemia among pregnant women in Dhaka city. BMC Women's Health.

[19] Ganle JK, Otupiri E, Parker M, Fitpatrick R (2015). Socio-cultural barriers to accessibility and utilization of maternal and newborn healthcare services in Ghana after user-fee abolition. International Journal of Maternal and Child Health.

[20] Ganle JK, Obeng B, Segbefia YA, Mwinyuri V, Yeboah YJ, Baatiema L (2015). How intra-familial decision-making affects women?s access to, and use of maternal healthcare services in Ghana: a qualitative study. BMC Pregnancy and Childbirth.

